# A triple-network organization for the mouse brain

**DOI:** 10.1038/s41380-021-01298-5

**Published:** 2021-10-14

**Authors:** Francesca Mandino, Roël M. Vrooman, Heidi E. Foo, Ling Yun Yeow, Thomas A. W. Bolton, Piergiorgio Salvan, Chai Lean Teoh, Chun Yao Lee, Antoine Beauchamp, Sarah Luo, Renzhe Bi, Jiayi Zhang, Guan Hui Tricia Lim, Nathaniel Low, Jerome Sallet, John Gigg, Jason P. Lerch, Rogier B. Mars, Malini Olivo, Yu Fu, Joanes Grandjean

**Affiliations:** 1 Singapore Bioimaging Consortium, Agency for Science, Technology and Research, 11 Biopolis Way, Singapore, 138667 Singapore; 2 Faculty of Biology, Medicine and Health, The University of Manchester, Manchester, UK; 3 Department of Radiology and Bioimaging Sciences, Yale School of Medicine, New Haven, CT USA; 4 Donders Institute for Brain, Cognition and Behaviour, Radboud University Medical Center, Nijmegen, The Netherlands; 5 Centre for Healthy Brain Aging, CHeBA, School of Psychiatry, University of New South Wales Medicine, Kensington, Sydney, NSW 2052 Australia; 6 Neurosurgery Service and Gamma Knife Center, Centre Hospitalier Universitaire Vaudois, Lausanne, Switzerland; 7 Wellcome Centre for Integrative Neuroimaging, Centre for Functional MRI of the Brain (FMRIB), Nuffield Department of Clinical Neurosciences, John Radcliffe Hospital, University of Oxford, Oxford, UK; 8 Department of Medical Biophysics, University of Toronto, Toronto, ON Canada; 9 Centre for Research and Development in Learning, Nanyang Technological University, 61 Nanyang Drive, Level 1, Singapore, 637460 Singapore; 10 University College London Medical School, University College London, London, UK; 11 Univ Lyon, Université Lyon 1, Inserm, Stem Cell and Brain Research Institute U1208, 69500 Bron, France; 12 Donders Institute for Brain, Cognition and Behaviour, Radboud University Nijmegen, Nijmegen, The Netherlands; 13 Department of Radiology and Nuclear Medicine, Radboud University Medical Center, Nijmegen, The Netherlands

**Keywords:** Neuroscience, Physiology

## Abstract

The triple-network model of psychopathology is a framework to explain the functional and structural neuroimaging phenotypes of psychiatric and neurological disorders. It describes the interactions within and between three distributed networks: the salience, default-mode, and central executive networks. These have been associated with brain disorder traits in patients. Homologous networks have been proposed in animal models, but their integration into a triple-network organization has not yet been determined. Using resting-state datasets, we demonstrate conserved spatio-temporal properties between triple-network elements in human, macaque, and mouse. The model predictions were also shown to apply in a mouse model for depression. To validate spatial homologies, we developed a data-driven approach to convert mouse brain maps into human standard coordinates. Finally, using high-resolution viral tracers in the mouse, we refined an anatomical model for these networks and validated this using optogenetics in mice and tractography in humans. Unexpectedly, we find serotonin involvement within the salience rather than the default-mode network. Our results support the existence of a triple-network system in the mouse that shares properties with that of humans along several dimensions, including a disease condition. Finally, we demonstrate a method to humanize mouse brain networks that opens doors to fully data-driven trans-species comparisons.

## Introduction

Brain disorders are defined by altered patterns of brain structure and function, which provide insights into their underlying mechanisms [1, 2]. The neuroimaging correlates for neuropsychiatric disorders have been shown to cluster into overlapping brain areas [3–5]. The triple-network model of psychopathologies proposes a theoretical framework based on the interactions within and between three networks to explain psychiatric and neurological traits [1]. The model encompasses three networks: the default-mode, salience, and central executive networks. These are defined by their characteristic spatial (overlapping areas) and temporal (e.g., default-mode and central executive networks’ anti-correlation) properties [6–8].

These networks are not unique to the human brain. Putative homologous networks have also been reported in nonhuman primates and rodents [9–12]. Determination of the underlying pathological mechanisms often relies on animal models, where invasive manipulations and measurements reveal actionable targets for the development of new therapies. Hence, the characterization of these networks in animals allows for translatable insights applicable to humans. Between-species comparisons rely on spatial assumptions of equivalency, yet these remain non-trivial to determine [13]. Here, we seek to characterize network homologies using a data-driven approach. To forgo approximate matching between species, we developed a new method to convert brain maps using spatial transcriptomic similarity between the mouse and the human brain [14, 15]. We propose that homologous brain states exist in three mammalian species commonly used in neuroscience research, namely human, macaque and mouse. To validate this, we generated humanized synthetic brain states derived from mouse brain maps and spatially compared them to matched human brain states. Beyond spatial homology, we hypothesize that transitions between brain states are conserved between the species examined, thus, defining temporal similarity.

In this work, we establish shared spatio-temporal brain state properties between the human, macaque, and mouse brains. We test the predictions of the triple-model in mice by examining brain state occurrences in a dataset containing resting-state recordings in a mouse model for depression [16]. We then generate a precise neuroanatomical model for the triple-network organization using a high-resolution mouse tracer dataset. Finally, we validate this anatomical model using functional stimulation of two key nodes of the salience network commonly implicated with depressive disorders, namely the dorsal raphe (DR) nucleus and the insular area. Using a combination of optogenetic stimulation during behavior and whole-brain imaging, we identify interactions between elements of the salience and default-mode networks associated with positive valence and cross-reference this with a meta-analysis of the human neuroimaging literature. Taken together, we demonstrate that triple-network model predictions for depressive traits apply in the mouse. Last but not least, our neuroanatomical model suggests a revision of the monoamine contribution in the regulation of these networks.

## Method

### Data and code availability

The complete preprocessed mouse functional Magnetic Resonance Imaging (fMRI) datasets are freely available at 10.34973/1he1-5c70 (resting-state) and 10.34973/raa0-5z29 (optogenetics, CAPs). The S1200 human connectome dataset is available at https://db.humanconnectome.org/ [17]. The viral tracer data is available from https://connectivity.brain-map.org/ [18]. The mouse template is available at https://atlas.brain-map.org/. The complete code to reproduce the study is freely available as an R notebook at https://gitlab.socsci.ru.nl/preclinical-neuroimaging/insula. The repository also includes tables, figures, and imaging assets. The code to produce synthetic brain maps is available at https://gitlab.socsci.ru.nl/preclinical-neuroimaging/homologous-brain-expression-map.

### Data processing and analysis

Mouse and macaque data were preprocessed as follows. Anatomical images were corrected for B1 field (*N4BiasFieldCorrection*), denoised (*DenoiseImage*), brain-masked (*antsBrainExtraction.sh*) and registered using SyN diffeomorphic estimation (*antsRegistration*) to the AIBS template (mouse, https://atlas.brain-map.org/) or F99 template (macaque) [19] using ANTs [20]. Functional images were despiked (*3dDespike*), motion-corrected (*3dvolreg*), corrected for B1 field, denoised, and brain-masked, before being registered linearly to their corresponding anatomical images. Resting-state fMRI (rsfMRI) data were band-pass filtered (*3dBandpass*, 0.01–0.25 Hz for the mouse, 0.01–0.10 Hz for the macaque). Optogenetic fMRI (ofMRI) data were high-pass filtered (0.01 Hz). Independent component analysis (*melodic*) was applied, and nuisance components were determined using FIX before being regressed out (*fslregfilt*). Z-statistics for evoked activity (ofMRI) or functional connectivity (rsfMRI) were determined using *fsl_glm*. The code to preprocess the mouse and macaque datasets is available at https://github.com/grandjeanlab/MouseMRIPrep.

### Brain state analysis

Brain states were derived using co-activation patterns (CAPs). These were estimated using the *TbCAPs* toolbox (https://c4science.ch/source/CAP_Toolbox.git) [21]. The anesthetized mouse dataset consisted of *N*_baseline_ = 47, *N*_CSS_ = 25, and *N*_control_ = 27 scans acquired at 9.4 T with repetition time = 1 s for 360 volumes [16]. Mice were mechanically ventilated and anesthetized with 0.5% isoflurane, together with 0.05 mg/kg medetomidine bolus and 0.1 mg/kg infusion (see *Optogenetics: imaging* for detailed protocol). The awake mouse dataset consisted of *N* = 54 scans, acquired at 11.75 T with repetition time = 1.2 s for 180 volumes. The macaque dataset consisted of 10 male rhesus macaques (median age = 4.98 years; median weight = 9.25 kg) imaged at 3 T under isoflurane (1%) anesthesia and acquired with repetition time = 2.28 s for 1600 volumes. The human rsfMRI dataset consisted of 15 humans imaged at 7 T in the awake condition with open eyes, sampled from the S1200 database [17], and was acquired with repetition time = 0.78 s for 900 volumes. Volume selection was performed according to two criteria: (1) activity within at least one of three seed regions—the insula, anterior cingulate area (ACA), and the dorsolateral prefrontal cortex (human), frontal eye field (macaque), or primary motor area (mouse), and (2) framewise displacement <0.5 mm for macaques and humans or 0.2 mm for mice [22]. This led to 80.4% (min = 73.9%, max = 85.3%) retained volumes on average in anesthetized mice, but 35.8% (min = 1,1%, max = 76.7%) in awake mice due to increased movement. In macaques, the percentage of retained volumes was lower (mean = 19.0%, min = 0.1%, max = 53.9%) than in humans (mean = 73.7%, min = 65.9%, max = 83.0%). The optimal K-means clustering dimension was estimated in anesthetized mice, macaques, and humans using consensus clustering [23] from sub-samples of 80% of volumes, and the percentage of ambiguously clustered pairs [24] was used as a quality criterion. An optimum was found for *N*_cluster_ = 6 in mice, *N*_cluster_ = 5 in macaque, and *N*_cluster_ = 6 in humans. We selected *N*_cluster_ = 6 across species, for comparability and in accordance with prior descriptions in anesthetized mice [25] and humans [26]. Co-activation pattern analysis operates through K-means clustering applied on the retained volumes across the whole analyzed population. Each CAP is one of the output centroids of the algorithm, that is, an average across the fMRI volumes clustered together. Since each volume is strictly associated with only one CAP, transition probabilities can be computed by quantifying how often a particular switch from the expression of a given CAP at time *t* to that of another CAP at time *t* + *1* occurs (note that if the same CAP is expressed in two consecutive frames, it counts as a transition of the CAP onto itself). The obtained normalized transition matrices, denoting this information for all possible pairs of CAPs, were vectorized and compared between species with linear models. Brain states were initially sorted between species using previously reported networks in nonhuman primates and in rodents [9–12].

### Synthetic brain maps

Synthetic brain maps were generated using in situ transcriptomic similarity between available mouse in situ hybridization and human microarray data. A list of mouse and human homolog genes was obtained using Ensemble Biomart (http://www.ensembl.org) and cross-referenced to the list of genes available in the Allen Brain Atlas (https://mouse.brain-map.org/). The human data (https://human.brain-map.org/) was downloaded from the Allen Brain Atlas and processed using the automatic pipeline from the abagen (v. 0.0.7) package [27]. In total, 3218 genes available in both 81 human brain regions-of-interest from the Desikan Killiany atlas [28] and mouse in situ whole-brain maps were retained. All genes were normalized to a range of 0–1 to account for amplitude differences. Mouse brain state maps derived from the CAP analysis were modeled as a linear addition of normalized transcriptomic in situ maps using a linear model. The parameter estimates for each of the 3218 genes were applied to the human gene array and reconstructed into a 3D map overlapping with the Desikan Killiany atlas. Synthetic brain maps were compared to matched and mismatched brain states using a spatial correlation based on vectors extracted with the Desikan Killiany atlas.

### Projection input similarity

Anterograde viral tracer maps (*N* = 498) obtained from injections into the right hemisphere in C57BL/6 male mice were obtained using the Allen Institute for Brain Science application programming interface and converted to NIFTI format in R using the ‘nat’ and ‘oro.nifti’ packages. Projection input similarity was estimated by extracting the tracer density profile from a seed region contralateral to the injection sites across all tracer maps and cross-referencing this profile to that of all other brain voxels. This effectively reveals regions that receive inputs from similar areas as the seed region, rather than comparing output projections from a seed region as in [29].

### Diffusion tensor imaging

The S1200 7T diffusion tensor imaging dataset (*N* = 178) was reconstructed using *‘bedpostx’* with default parameters. Probabilistic tractography was performed using *‘probtrackx’*, also with default parameters. Three seeds positioned on the left hemisphere were used: anterior insular area (MNI152 coordinates −36.14, 10.30, 2.56), ACA (−6.77, 45.80, 21.12), and dorsolateral prefrontal cortex (−43.83, 25.41, 35.44), for the salience, default-mode, and central executive networks, respectively. Seed positions were informed from the co-activation pattern analysis. Gray-matter projections were extracted using *‘fslmeants’* and the Desikan Killiany atlas, and spatially compared to humanized synthetic brain maps.

### Optogenetics: animal permit

All applicable international, national, and institutional guidelines for the care and use of animals were followed. All procedures performed in studies involving animals were in accordance with the ethical standards of the Institutional Animal Care and Use Committee (A*STAR Biological Resource Centre, Singapore, IACUC #161134). A total of *N* = 18 mice were used to acquire de novo data in this study.

### Optogenetics: surgery

Mixed-sex mice (~14–18 weeks, ~25 g) were anesthetized with a mixture of ketamine/xylazine (ketamine 75 mg/kg, xylazine 10 mg/kg). The head was shaved and cleaned with three wipes of Betadine^®^ and ethanol (70%). Lidocaine was administered subcutaneously, *in loco*. Each animal was kept on a warm pad to maintain normal body temperature. The head was positioned in a stereotaxic frame and protective ophthalmic gel was applied to avoid dryness of the eyes. A portion of the scalp was removed to expose the skull. The skull was perforated with a drill (burr tip Ø 0.9 mm^2^). AAV5-CaMKIIa-hChR2(H134R)-mCherry (*N* = 10, 0.75 μl, titer 1–8 × 10^12^ vg/ml, Vector Core at the University of North Carolina, Chapel Hill) or AAV5-CaMKIIa-mCherry (*N* = 8) controls were injected at +1.8 mm Anterior-Posterior, +2.5 mm Medio-Lateral, −2.0 mm Dorso-Ventral relative to Bregma into the left hemisphere, corresponding to the agranular insular area. A fiber-optic cannula (Ø 200 μm, 0.39 NA, length according to the injection site, Ø 1.25 mm ceramic ferrule) was lowered to the target region (Laser 21 Pte Ltd, Singapore; Hangzhou Newdoon Technology Co., Ltd, China). The cannula was fixed in place with dental cement (Meliodent rapid repair, Kulzer). Buprenorphine was administered post-surgically to each animal. Animal recovery took place on a warm pad.

### Optogenetics: imaging

Animal preparation was performed as described previously [30]. Anesthesia was induced with 4% isoflurane; subsequently, the animals were endotracheally intubated, placed on an MRI-compatible cradle, and artificially ventilated (90 breaths/minute; Kent Scientific Corporation, Torrington, Connecticut, USA). A bolus with a mixture of medetomidine (Dormitor, Elanco, Greenfield, Indiana, USA) and Pancuronium Bromide (muscle relaxant, Sigma-Aldrich Pte Ltd, Singapore) was administered subcutaneously (0.05 mg/kg), followed by a maintenance infusion (0.1 mg/kg/h) while isoflurane was simultaneously reduced and kept to 0.5%. Care was taken to maintain the animals’ temperature at 37 °C using a feedback-controlled water bath. The data were acquired on an 11.75 T scanner (Bruker BioSpin MRI, Ettlingen, Germany) equipped with a B-GA09S gradient system, a 72 mm linear volume resonator coil for transmission, and a 10 mm single loop surface coil. Images were acquired using Paravision 6.0.1 software. An anatomical reference scan was acquired using a spin-echo Turbo-RARE sequence: field of view (FOV) = 17 × 9 mm², FOV saturation slice masking non-brain regions, number of slices = 21, slice thickness = 0.45 mm, slice gap = 0.05 mm, matrix dimension (MD) = 200 × 100, repetition time (TR) = 2742 ms, echo time (TE) = 30 ms, RARE factor = 8, number of averages = 2. fMRI was acquired using a gradient-echo echo-planar imaging sequence with the same geometry as the anatomical scan: MD = 60 × 30, TR = 1000 ms, TE = 11.7 ms, flip angle = 50°, volumes = 720, bandwidth = 119047 Hz. Field inhomogeneity was corrected using MAPSHIM protocol. ChR2 photostimulation was provided through a blue light laser (473 nm, LaserCentury, Shanghai Laser & Optic Century Co., Ltd; ~12–15 mW output with continuous light at the tip of the fiber). After an initial 50 s of rest as a baseline, 10 s of 20 Hz 10 ms light pulses were followed by 50 s of rest period, in a 10-block design fashion. An additional 60 s of rest was recorded after the last block of stimulation. Areas of photostimulation were estimated based on lesions caused by the optic fiber, as revealed with anatomical images. The photostimulation area was modeled using the default brain tissue parameters in Doric Neuroscience Studio (Doric Lenses Inc, Quebec, Canada).

### Literature meta-analysis

Literature meta-analysis was performed using the neuroquery toolbox [31] for the following terms: ‘depression’ (https://neuroquery.org/query?text=depression+, query date: 10/07/2020) and ‘positive valence’ (https://neuroquery.org/query?text=positive+valence+, query date: 09/06/2020). Both queries returned 78 entries, which are represented as ‘overlap maps’, with the default threshold applied by the neuroquery toolbox.

### Statistics and data representation

Voxel-wise one-sample and two-sample *t*-tests were determined using *fsl_glm*, and cluster-corrected for multiple comparisons using the *easythresh* function, with a threshold set at either *p* = 0.05 or 0.001, as specified under the color bar. Linear models are estimated in R using the ‘*lm*’ function. Standardized coefficient parameters and 95% confidence intervals are estimated using the ‘parameters’ package for R [32]. Group comparisons are represented using Gardner-Altman plots (https://www.estimationstats.com/#) [33]. The raw data are plotted on the left or upper axes; each mean difference is plotted on the right or lower axes as a bootstrap sampling distribution. Group differences are normalized to Cohen’s d. These are depicted as dots; 95% confidence intervals are indicated by the ends of the vertical error bars. Inferences are made on the standardized coefficient and/or Cohen’s d, together with their 95% confidence intervals, following the Cohen 1988 interpretations (small: <0.2; medium: <0.4; large: <0.8) [34].

## Results

**Fig. 1:**
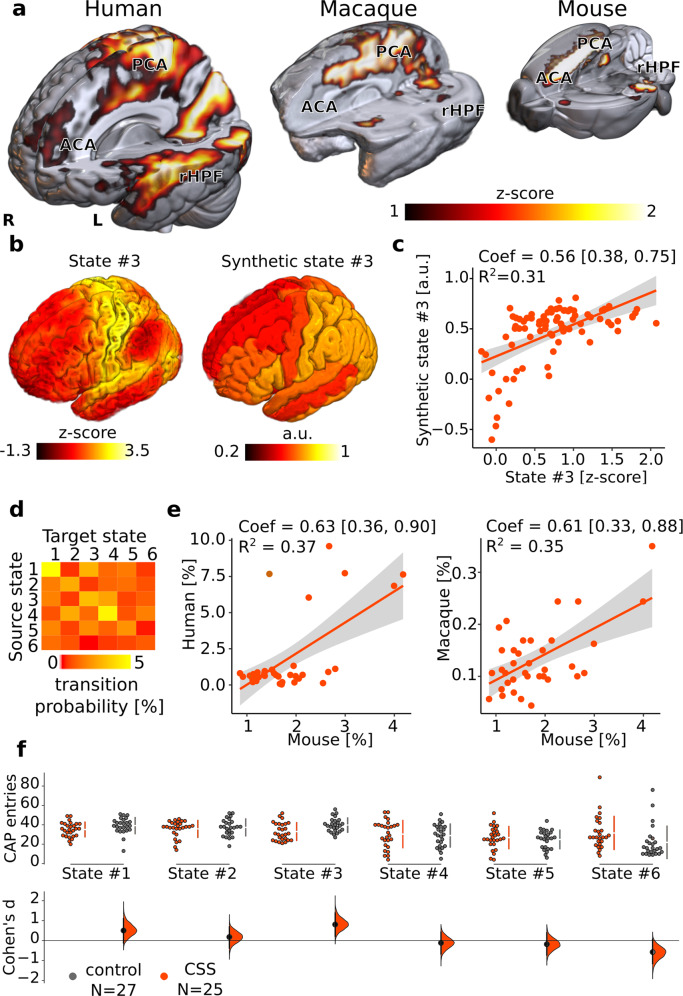
Functional network homologies in three mammalian species. **a** Brain state #3 exhibits default-mode-like overlap in human, macaque, and mouse brains (see Fig. S1 for details). **b** 3D-rendered state #3 (left) and its synthetic map (right) obtained from the matched mouse brain state. **c** Spatial correlation between state #3 and its matching synthetic map. Lines and ribbons indicate the regression lines and 95th confidence intervals, respectively (see Fig. S3 for details). **d** State transition probability matrix in the mouse. **e** Correlated transition probabilities between the mouse and the human (left) or macaque (right). **f** Number of state entries in a chronic social stress dataset (*N*_CSS_ = 25, orange; *N* = 27_control_, gray). Cohen’s d and bootstrapped 95th confidence interval (bottom) between dataset (top). ACA: Anterior cingulate area, PCA: posterior cingulate area, rHPF: retro-hippocampal formation, CSS: chronic social stress.

To compare resting brain activity patterns in the three species, we reduced resting-state functional Magnetic Resonance Imaging (fMRI) fluctuations acquired in human, macaque, and mouse recordings into brain states, the CAPs [21]. The human data consisted of a sub-set (*N* = 15 subjects) of the 7 Tesla Human Connectome Project S1200 dataset [17]. The macaque data (*N* = 10) was acquired in free-breathing anesthetized macaques [35]. The mouse data (*N* = 47) consisted of the baseline scans of ventilated mice previously acquired [16]. We clustered the images acquired in humans, macaques and mice into six brain states consisting of a spatial map and a temporal activation profile [21, 36]. We opted for six states as this was the optimal clustering value for the mouse dataset, and was also reported as such, previously [25]. Advantageously, such a representation of brain activity is data-driven, comprehensive, and comparisons can be made in absence of exact spatial homology. We observed matching spatial patterns across species (Figs. 1a, S1). The states mainly described co-(de)activation clusters overlapping with areas previously associated with the default-mode (#1, 2, 3), the salience (#5, 6), and the central executive network (#1, 2, 4), referred to as the lateral cortical network in mice [9]. For instance, state #3 displays co-activation in the anterior and posterior cingulate areas (ACA, PCA. respectively), and retro-hippocampal formation (rHPF), characteristic of the default-mode network (Fig. 1a). To account for differences between awake and anesthesia contributions, we reproduced the brain states in a dataset obtained with awake mice (Fig. S2), demonstrating brain state stability across physiological conditions. We concluded that homologous brain states exist in all three species examined.

Brain states were first matched between species based on spatial homology with previously described networks [9, 12]. To obtain a data-driven comparison, we generated synthetic human brain states from the mouse maps. Mouse brain states were vectorized and modeled as the weighted sum of 3218 gene expression maps obtained from the in situ hybridization mouse brain database [15]. The parameter estimate for each mouse gene expression was multiplied with the homology-matched human gene expression derived from the microarray human brain database [14], and summed to obtain synthetic brain maps. Matched human brain states and corresponding synthetic maps exhibited greater spatial correlations than mismatched maps (Fig. 1b, c, synthetic_state #3_ ~ matched_state #3_: Coef = 0.56 [0.38, 0.75], Fig. S3, matched minus mismatched: Cohen’s d = 0.72 [−0.02, 1.75]). This confirmed our qualitative matching.

Beyond spatial homology, we sought to identify temporal homology between species. Specifically, we hypothesized that transitions between states are non-random and conserved between species. By projecting brain states into the resting-state data, we obtained state transition probabilities, as well as the number of entries into each of the six states. We found that the relative state transition probabilities between matched states were correlated between species (Fig. 1d, e, Transition_Human_ ~ Transition_Mouse_: Coef = 0.63 [0.36, 0.90], Transition_Macaque_ ~ Transition_Mouse_: Coef = 0.61 [0.33, 0.88]). This suggests that temporal dynamics between these brain states are conserved.

Having established common spatio-temporal patterns between mice, macaques, and humans, we sought to establish if triple-network predictions about disease mechanisms would translate to mice. We hypothesized that, similar to humans (Fig. S4), both the default-mode and salience networks would be affected in a mouse model for depression [16, 37, 38]. We projected the brain states onto the resting-state fluctuations acquired in mice that underwent chronic social stress (CSS, *N*_CSS_ = 25; *N*_control_ = 27), a paradigm to induce depression-relevant behavioral traits [37, 38]. Consistent with our hypotheses, state #3, overlapping with the default-mode, was entered more frequently in the stressed mice relative to controls (Fig. 1f, CSS minus control: Cohen’s d = 0.80 [0.21, 1.38]), while state #6, overlapping with a deactivated salience network, was visited less often (Cohen’s d = −0.58 [−1.15, 0.06]). The brain states affected in our chronic stress model overlapped with networks implicated in depression in humans as predicted by the triple-network model and were confirmed with our meta-analysis. This similarity suggests that the mechanisms underlying brain state dysregulation in animal models of stress may translate to humans. Moreover, the presence of this phenomenon in lightly anesthetized mice suggests that this effect is hardwired rather than context-dependent.

**Fig. 2:**
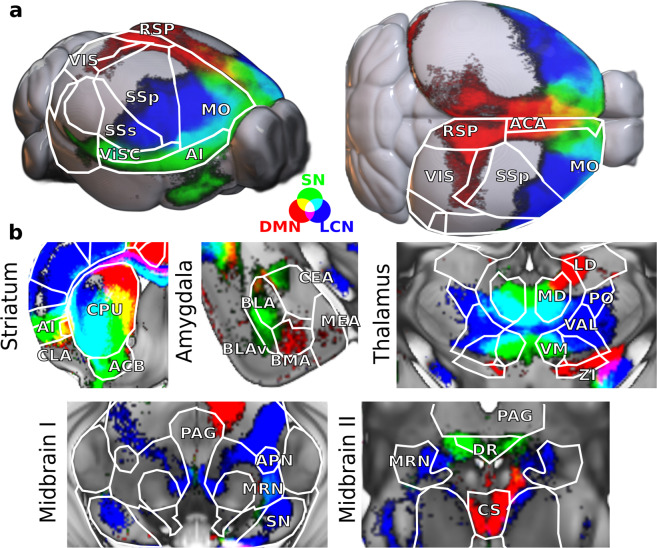
A network model for the mouse. Projection input similarity in the cortex (**a**) and subcortical areas (**b**) delineate areas between the salience (SN, green), default-mode (DMN, red), or lateral cortical network (LCN, blue). Color-coded statistical maps are thresholded at *p* ≤ 0.05, cluster-corrected. Extended slices are presented in Fig. S5a.

Brain states and the functional networks that they encompass are anchored into a structural network consisting of local connections and distal projections [29, 39, 40]. Viral tracer methods remain the gold standard to establish structural networks at a high resolution [18], but are not applicable in humans. We sought to characterize the anatomical substrate underlying the network dynamics in the mouse brain. Contrary to previous endeavors that compared functional connectivity against projection outputs, we examined the projection input similarity. This framework allows for a more direct comparison to functional neuroimaging due to a common analysis using seeds. Projection input similarity for the default-mode, salience, and lateral cortical networks was determined relative to three seeds: the ACA, agranular insula (AI), and primary motor areas (MOp). This analysis showed that these three networks are anchored into chiefly non-overlapping cortical (Fig. 2a) and subcortical territories (Figs. 2b, S5a). These patterns were also recapitulated in the corresponding functional maps, consistent with the notion of structurally supported functional networks (Fig. S6). In addition to well-established cortical delineations, we generated detailed subcortical sub-divisions of the three networks (Fig. 2b). The striatum is divided across the dorsal (default-mode), lateral (lateral cortical), and ventral (salience) axes. The amygdaloid area elongates across the salience and the default-mode networks, with basolateral (BLA) and central amygdala in the former and medial (MEA) and basomedial amygdala in the latter network. The default-mode network also encompassed the zona incerta (ZI) and lateral-dorsal nucleus (LD), whereas the lateral cortical network included the ventral posterior part of the thalamus (VM). In the midbrain, the substantia nigra (SN) was associated with the lateral cortical, while the serotonin-releasing DR and the superior central raphe (CS) were part of the salience and default-mode networks, respectively. This detailed neuroanatomical model echoes similar work in humans achieved with diffusion imaging [41], albeit with enhanced detail and nucleus specificity.

Importantly, we observe a clear network division between the raphe nuclei (dorsal vs. central). This result suggests a revision to models for monoamine involvement in human networks that have associated the serotoninergic system with the default-mode network exclusively [42, 43]. To validate this neuroanatomical model, we humanized the three tracer-based structural networks using the conversion method detailed above. We compared gray matter overlap with the projections estimated from probabilistic tractography in a diffusion tensor imaging dataset (*N* = 178). Both the humanized default-mode and lateral cortical network grey matter loadings were strongly associated with the tractography-estimated projections (Fig. S5b, Synthetic_DMN_ ~ Tractography_DMN_: Coef = 0.34 [0.08, 0.60], Synthetic_LCN_ ~ Tractography_CEN_: Coef = 0.60 [0.27, 0.94]). The humanized mouse default-mode structural network recapitulated the key features of the human network, namely hyperintensity in the ACA, PCA, as well as medial temporal lobes (Fig. S5c). We conclude, therefore, that the mouse structural networks recapitulate features expected in humans. Further, we posit that the fine subcortical neuroanatomical details established in the mouse also translate to the human brain.

**Fig. 3:**
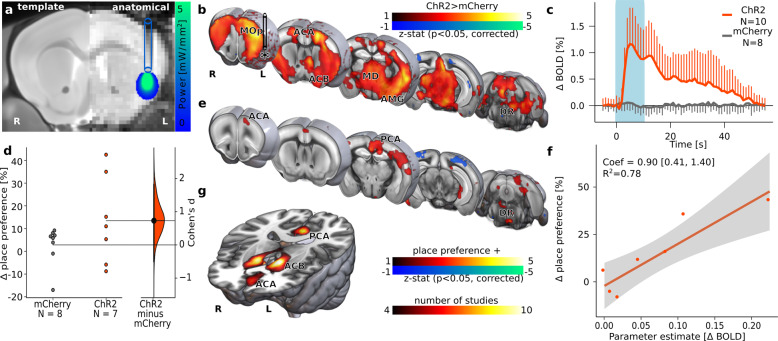
Optogenetics photostimulation of the insular area. **a** Modeled illumination of the insula. **b** Evoked response to photostimulation blocks in ChR2 (*N* = 10) vs. mCherry (*N* = 8) controls. The asterisk indicates the photostimulation site. **c** Averaged insula BOLD response to photostimulation blocks. Error bars indicate ±1 standard deviation. **d** Conditioned place preference induced by photostimulation. **e** BOLD response correlated with place preference in ChR2-injected mice. **f** BOLD parameter estimates in the posterior cingulate area as a function of place preference. The line and ribbon indicate the regression line and 95th confidence interval, respectively. **g** Literature meta-analysis for the search term ‘positive valence‘.

Understanding the functional contribution of these networks is central to our understanding of psychopathologies. Hence, we tested the functional integration of two nodes within the salience network implicated in depressive disorders, namely the DR nucleus and insular area. We modeled the distributed response to neuronal perturbations evoked by channelrhodopsin-2 (ChR2) photostimulation as a function of the neuroanatomical maps for the three networks (Fig. 2). First, we examined the changes in cerebral blood volume in an fMRI dataset where optogenetic (ChR2, *N* = 63 runs) photostimulation was performed on ePet-positive DR neurons relative to yellow fluorescent protein (YFP, *N* = 18 runs) controls [44]. The statistical map resulting from the comparison chiefly overlapped with that of the salience network (Fig. S7ac, Tracer_AI_ ~ CBV response: Coef = 0.20 [0.09, 0.30]), consistent with the indication that the DR nucleus is embedded into the salience structural network. The second node targeted consisted of CaMKII-positive neurons in the insular area. Similar to DR ePet-positive neurons, we found that photostimulation of CaMKII-positive neurons in the insula (*N* = 10) elicited a response overlapping with the salience network relative to mCherry-transfected controls (*N* = 8, Tracer_AI_ ~ BOLD response: Coef = 0.24 [0.14, 0.35], Figs. 3a–c, S7bd, S8c). In agreement with the pivotal role of the insula in regulating activity within lateral cortical and default-mode networks [1], photostimulation of its neurons also revealed elements within these networks, respectively the primary motor areas and ACA. Given the association of the insula with the nucleus accumbens (ACB), a hub for reward, and the role of the salience network in valence mapping, we hypothesized that photostimulation of the insula would lead to positive valence, resulting in conditioned place preference. Aberrant valence mapping is associated with the induction and maintenance of depression [45]. However, we could not conclude that insula photostimulation leads to positive valence (Fig. 3d, ChR2 minus mCherry: Cohen’s d = 0.73 [−0.49, 1.83]). The variability in the evoked behavior in the ChR2 group could be due to individual differences in network engagement to photostimulation. To test this, we regressed the place preference parameter into individual functional maps. This revealed clusters in the ACA, PCA, both positively associated with place preference (Fig. 3e, f, Δplace preference ~ PCA**:** Coef = 0.90 [0.41, 1.40]). To test how cingulate involvement in positive valence translates to humans, the optogenetic response was compared to a meta-analysis for the search term ‘positive valence’ in human neuroimaging studies (Fig. 3g). There was an elevated overlap in the literature in association with ‘positive valence’ in both ACA, PCA. These loci are also highly associated with the default-mode network and they are areas highlighted in our ‘depression‘ meta-analysis (Fig. S4). We conclude that specific network engagement between the salience and default-mode networks enhances the conditioning for place preference.

## Discussion

We demonstrate that resting-state fluctuations consist of dynamically occurring brain states that are conserved across three species, and encode pathological manifestations, such as depression-relevant conditions in mice. Further, we demonstrate a triple-network system in mice and the detailed anatomical subcortical substrate that supports it. Finally, we show that evoked activity within the nodes belonging to the salience network preferentially elicits activity within that network and that photostimulation of the insula elicits a positive valence when co-activation of cingulate areas is present. Taken together, we provide a model highlighting neuromodulation targets to control specific networks and to serve as a common currency to translate findings between neurobiological observations in rodents and network dynamics assessed in humans with neuroimaging.

We demonstrate the existence of homologous brain states in the human, macaque, and mouse brains. Unexpectedly, we find that the serotoninergic system is associated with different elements of the triple network; specifically, the DR was found to be structurally and functionally associated with the salience network. This contrasts with previous work in humans that has suggested an association with the default-mode network [42, 43]. Our observation is further supported by other studies in rodents that have shown the involvement of the ventral striatum, a node of the salience network, in response to psilocybin, a 5HT2a receptor agonist [46, 47]. Puzzlingly, this contrasts with a comparable study in humans that indicated default-mode network involvement instead [48] (but also a ventral striatum response [49]). Whether these network involvement inconsistencies are due to species differences, or to anesthesia, will need to be determined; however, viral tracers are independent of anesthesia, providing anatomical support for our hypothesis. Finally, it should be noted that the serotoninergic system extends beyond the dorsal and CS nuclei, with at least nine nuclei characterized and each containing heterogeneous neuronal populations [50]. The regulation of distributed neuronal networks is expected to be mediated by complex mechanisms.

This work is facilitated by the availability of large online databases for human and mouse rsfMRI, meta-analysis tools, diffusion imaging, viral tracers, and spatial transcriptomics [14, 15, 17, 18, 27, 31]. While we used nonhuman primate data as an intermediary for our first comparison, the relative lack of resources, such as spatial transcriptomics, highlights a need in the community for additional nonhuman primate resources to strengthen our comparisons [51, 52]. Fortunately, publicly available nonhuman primate resources are growing; this will allow for future comparisons across different modalities and scales between species, to better unravel the puzzling mechanisms leading to abnormal neuronal network function.

## Supplementary information

Supplemental material
