# Supplementary material for: A triple-network organization for the mouse brain
Source: Mol Psychiatry. 2021 Oct 14;27(2):865–72. doi: 10.1038/s41380-021-01298-5 (PMC9054663; doi:10.1038/s41380-021-01298-5)
Supplement: Supplementary file 1 — Supplemental material [file 41380_2021_1298_MOESM1_ESM.docx]

**Supplementary method**

*Histology*

After the completion of the experiments, animals were injected with an overdose of Ketamine/Xylazine and transcardially perfused with phosphate-buffered saline (PBS, 0.01 M) followed by 4% paraformaldehyde (PFA) in 0.01 M PBS. After extraction, the brain was post-fixed in 4% PFA overnight. Brain sections at 40 μm were made with a vibratome (CM3050S, Leica Microsystems, Nussloch, Germany), and fluorophore expression was assessed through a Ti-E inverted microscope (SBIC-Nikon Imaging Center, Singapore) for anatomical confirmation of viral injection and fiber optic cannula positioning.

*Slice electrophysiology*

Mouse brains were rapidly removed after decapitation and placed in high sucrose, ice-cold oxygenated artificial cerebrospinal fluid (ACSF) containing the following (in mM): 230 sucrose, 2.5 KCl, 10 MgSO4, 0.5 CaCl2, 26 NaHCO3, 11 glucose, 1 kynurenic acid, pH 7.3, 95% O_2_ and 5% CO_2_. Coronal brain slices were cut at a thickness of 250 μm using a vibratome (VT1200S, Leica Microsystems, Nussloch, Germany) and immediately transferred to an incubation chamber filled with ACSF containing the following (in mM): 119 NaCl, 2.5 KCl, 1.3 MgCl2, 2.5 CaCl2, 1.2 NaH2PO4, 26 NaHCO3, and 11 glucose, pH 7.3, equilibrated with 95% O_2_ and 5% CO_2_. Slices were allowed to recover at 32°C for 30 minutes and then maintained at room temperature for experiments. Whole-cell patch-clamp recordings were performed on AI pyramidal cells expressing ChR2-mCherry visualized using a CCD camera and monitor. Pipettes used for recording were pulled from thin-walled borosilicate glass capillary tubes (length 75 mm, outer diameter 1.5 mm, inner diameter 1.1 mm, WPI) using a DMZ Zeits-Puller (Zeitz). Patch pipettes (2–4 MΩ) were filled with an internal solution containing (in mM): 105 K-gluconate, 30 KCl, 4 MgCl2, 10 HEPES, 0.3 EGTA, 4 Na-ATP, 0.3 Na-GTP, and 10 Na2-phosphocreatine (pH 7.3 with KOH; 295 mOsm), for both voltage- and current-clamp recordings.

Photostimulation (460 nm) was delivered by an LED illumination system (pE-4000, CoolLED Ltd, UK). Several trains of square wave pulses (20 ms duration at 5 or 20 Hz) were delivered under current-clamp mode (I = 0) to examine whether neurons could follow high-frequency photostimulation. After different frequencies of photostimulation were completed, neurons were shifted to voltage-clamp mode (at -60 mV), and a prolonged square pulse of 500 ms duration was delivered, to further confirm whether ChR2-induced current could be seen in the recorded neurons. Access resistance, membrane resistance, and membrane capacitance were consistently monitored during each experiment to ensure the stability and the health of the monitored cell.

*Behavioral assay*

Conditioned place preference was recorded in a 50 x 35 cm plexiglass box with printed marks on the floor to serve as visual clues for the animals to identify the two compartments. The behavioural recording was performed using EthoVision XT12 software (Noldus Information Technology, Wageningen, the Netherlands). A 30 min baseline session with open compartments was performed to determine baseline place preference. In the following 3 days, the animals underwent 2 daily 30 min sessions in each closed compartment, one session in the morning and one in the afternoon. In the least preferred compartment, as determined during baseline, animals underwent repeated 10 s photostimulation blocks (20 Hz stimulation frequency, 10 ms pulse width) every 30 s. In the second compartment, an optic fiber was coupled to the head implant, but no photostimulation was applied. On the 5^th^ day, the animals underwent a 30 min test session. Δ place preference was determined as place preference for the photostimulation-associated compartment on test minus baseline sessions. 3 ChR2-transfected mice did not recover from the imaging session and could not be included in this experiment.


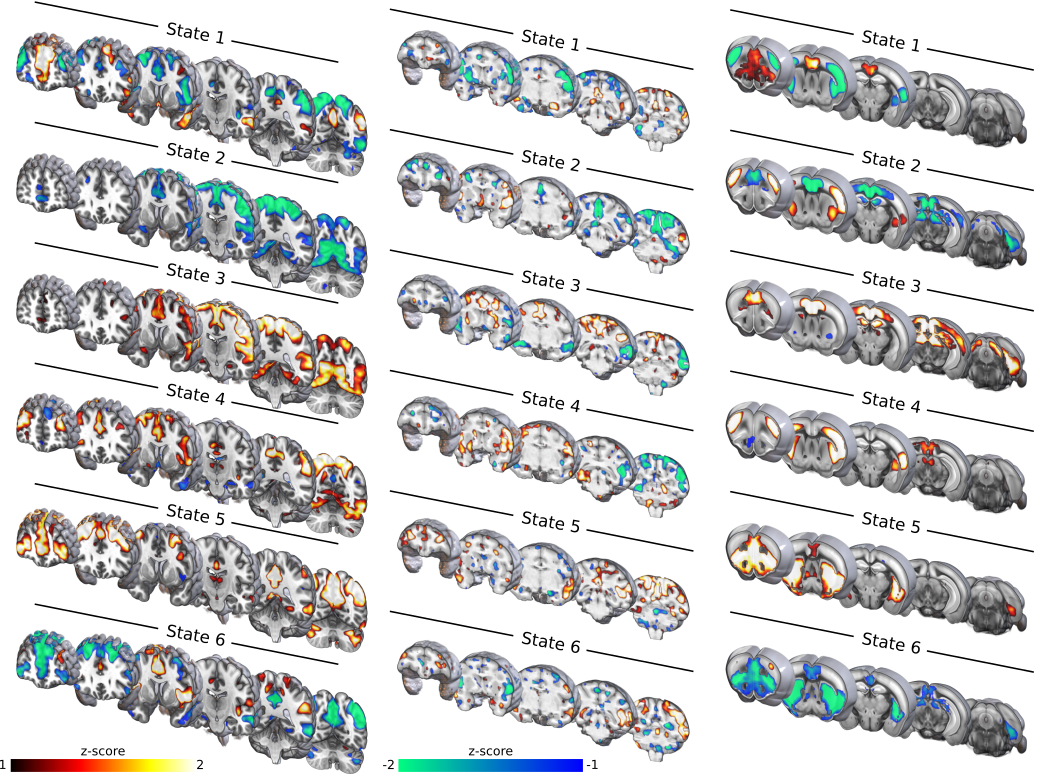


**Figure S1.** Brain states in humans (N = 15, left), isoflurane-anesthetized macaques (N = 10, middle), and medetomidine/isoflurane-anesthetized mice (N = 47, right). Human resting-state fMRI data were drawn from the 7T S1200 human connectome dataset, and pre-processed according to the human connectome pipeline, including FIX-denoising. Macaque resting-state fMRI data were obtained at 3T under ~1% isoflurane, and pre-processed with the same scripts as the mouse data. Brain states are matched according to spatial homologies across species, relative to the mouse. In the mouse, K-means clustering of resting-state fluctuations recapitulates previously described patterns [[1]](https://paperpile.com/c/qBPnxE/ZjRd).


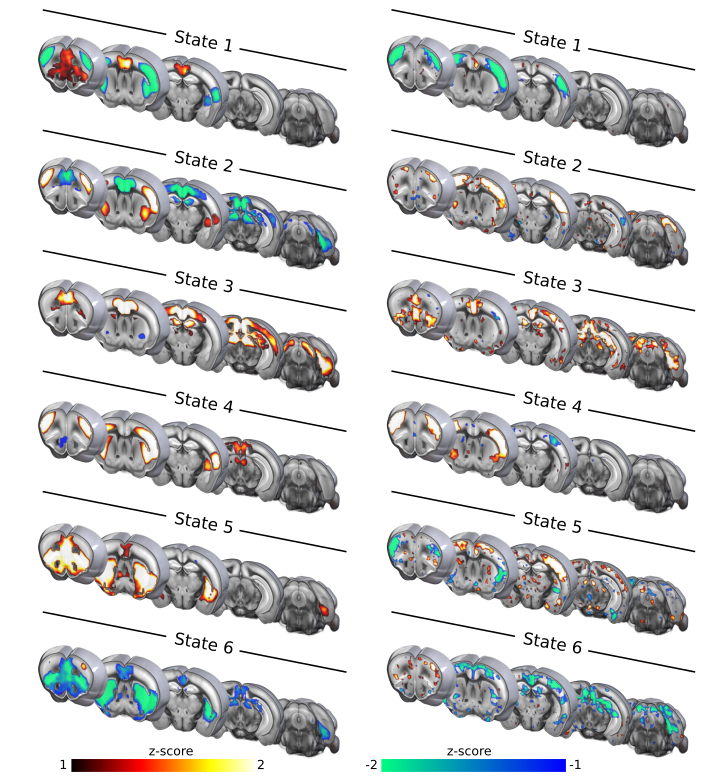


**Figure S2.** Brain states in medetomidine/isoflurane-anesthetized (N = 47, left) and awake (N = 54, right) mice indicate overlapping patterns in 3 out of 6 brain states. The awake dataset (10.18112/openneuro.ds001653.v1.0.1) consists of female C57BL/6 mice (12-15 weeks of age) trained for over 10 days, starting at 2 minutes for two days and subsequently at periods of 5, 10, 30, and 60 minutes. Each mouse was lightly anesthetized with isoflurane before restraining it by wrapping it in fabric, followed by taping it down to the cradle, and imaged with a cryogenic coil at 11.75T. Compromised overlap in the remaining states may be explained by the high number of frames removed due to excessive motion in the awake dataset (35.8% frames retained vs. 80.4% in the anesthetized dataset). Consequently, it remains difficult to infer the qualitative differences between the datasets.


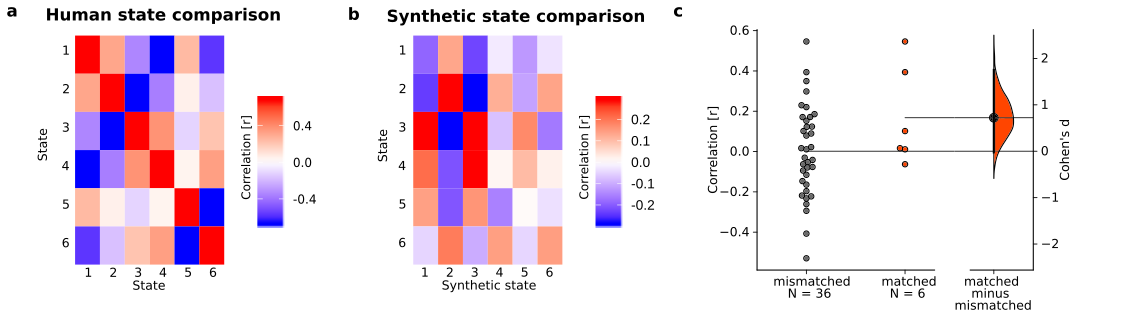


**Figure S3. a)** Spatial cross-correlation between human-derived brain states. Z-scores were extracted using the Desikan Killiany atlas containing 83 regions of interest. **b)** Spatial cross-correlation between human-derived brain states and mouse-derived synthetic brain maps. Synthetic brain maps were generated by converting mouse brain maps into a linear addition of weighted transcriptomic *in-situ* hybridization brain maps, and applying the weighted factors into homologous human brain gene transcription data. **c)** To assess the correspondence between the synthetic maps and the original human brain states, correlations between matched states (corresponding to the diagonal in **b**) are compared to mismatched states (above and below the diagonal in **b**). On average, matched synthetic maps and their corresponding states are more spatially correlated than mismatched maps (Cohen’s d = 0.72 [-0.02, 1.75]). This indicates plausible trans-species map conversions based on transcriptomic homology. The existence of strongly correlated synthetic and mismatched brain states is likely explained by strong (anti-)correlations between brain states (**a**), an observation previously reported in [[1]](https://paperpile.com/c/qBPnxE/ZjRd).

**
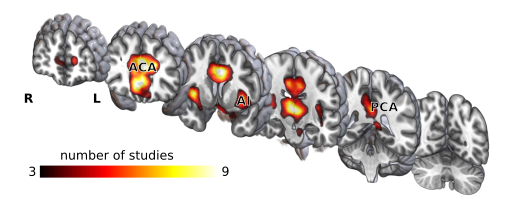
**

**Figure S4.** The triple-network model for psychopathology proposes that functional and structural endophenotypes for brain disorders cluster within three networks: the salience (SN), default-mode (DMN) and central-executive (CEN) networks. To test this assertion in the context of depression, we performed a neuroimaging literature meta-analysis for the search term `depression`. This reveals literature enrichment clusters in the anterior and posterior cingulate areas (ACA, PCA) and insula (AI), which are respectively elements of the DMN and SN. The involvement of these two networks is interpreted as DMN hyperactivity, associated with self-reference and rumination, due to altered valence mapping by the SN in depressive states [[2]](https://paperpile.com/c/qBPnxE/JCPP).

**
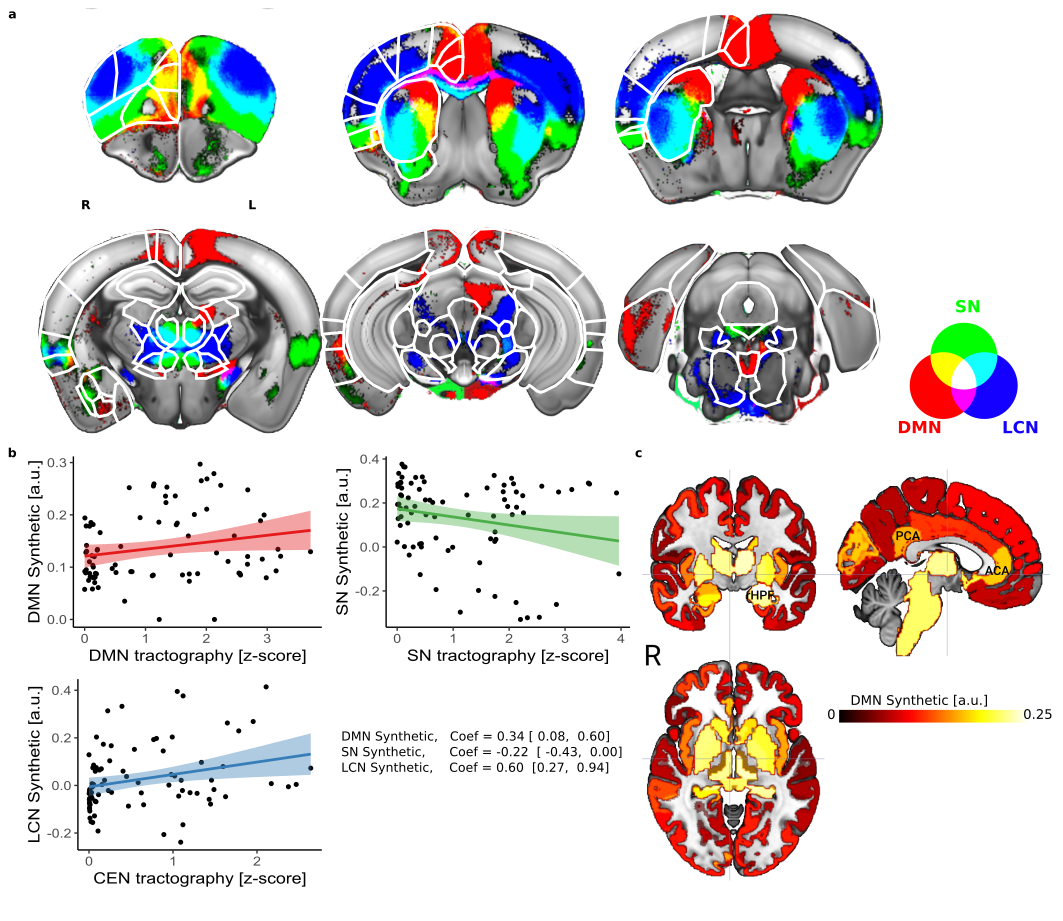
**

**Figure S5. a)** Full slices from excerpts shown in **Figure 2** showing projection input similarity relative to the insular area (SN, green), the anterior cingulate area (DMN, red), and the primary motor area (LCN, blue). Interestingly, no overlap between the three networks (indicated by the white color) is found except in white matter areas, reflecting axonal tracts. This shows that no structural network shares projection similarities with the insular, anterior cingulate and primary motor areas concurrently. Some areas demonstrated an overlap between two structural networks, e.g., the claustrum, which presents projection similarities with both the insular and anterior cingulate areas (yellow). **b)** Region-of-interest extracts for humanized tracer maps as a function of statistical maps derived from 7T Human Connectome Project Diffusion Tensor Imaging tractography. Both the humanized mouse default-mode and lateral cortical networks estimated with viral tracers overlap strongly with the counterparts estimated with tractography (Synthetic_DMN_ ~ Tractography_DMN_: Coef = 0.34 [0.08, 0.60], Synthetic_LCN_ ~ Tractography_CEN_: Coef = 0.60 [0.27, 0.94]). **c)** Humanized mouse default-mode network showing hyperintensity (warm colors) in anterior (ACA) and posterior cingulate areas (PCA), as well as the hippocampal formation (rHPF), areas associated with the human default-mode network. DMN: default-mode network; LCN: lateral cortical network; SN: salience network; CEN: Central executive network.

**
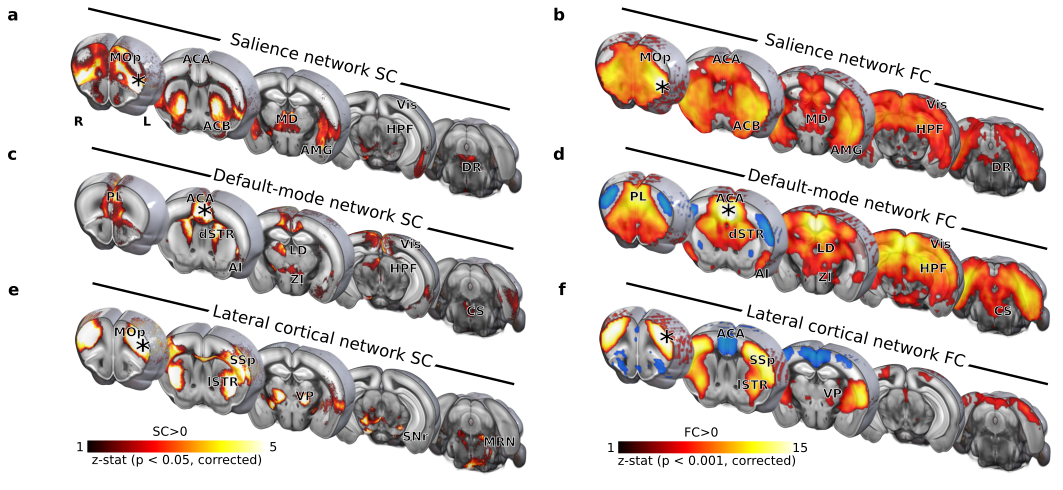
**

**Figure S6. a and b)** Structural and functional connectivity associated with the insular area (AI, asterisk) reveal the murine salience network. This includes the nucleus accumbens (ACB), insular area (AI), mediodorsal nucleus of the thalamus (MD), amygdala (AMG), and dorsal raphe nucleus (DR). Structural connectivity was determined using projection input similarity. **c and d)** Structural and functional connectivity associated with the anterior cingulate area (ACA, asterisk), reveal the murine DMN. This includes the prelimbic area (PL), dorsal striatum (dSTR), insular area (AI), dorsolateral nucleus of the thalamus (LD), zona incerta (ZI), visual area (Vis), hippocampal formation (HPF), and superior central raphe nucleus (CS). **e and f)** Structural and functional connectivity associated with the primary motor area (MOp) reveal co-activity with the primary somatosensory areas (SSp), lateral striatum (lSTR), and ventroposterior nucleus of the thalamus (VP). Significant anti-correlations are found in the ACA. Interestingly, functional networks expanded further than structural networks, consistent with the polysynaptic events captured with functional MRI [[3]](https://paperpile.com/c/qBPnxE/6DnJ). Maps were generated using one-sample t-tests, n = 498 viral tracer maps for structural connectivity, n = 186 scans for functional connectivity.


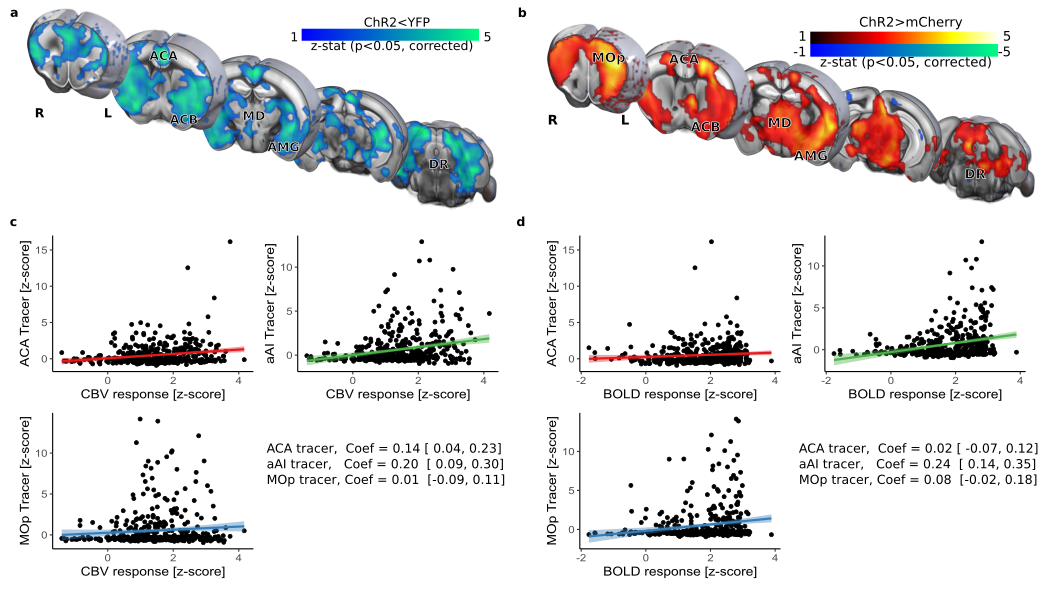


**Figure S7. a)** Cerebral blood volume decreases in response to Channelrhodopsin-2 photoactivation of the ePet-positive serotonergic neurons within the dorsal raphe (ChR2, N = 63 runs) relative to yellow fluorescent protein-transfected (YFP, N = 18 runs) controls.  **b)** BOLD increase in response to Channelrhodopsin-2 photoactivation of the CaMKII-positive neurons within the anterior insular area (ChR2, N = 10) relative to yellow fluorescent protein-transfected (mCherry, N = 8 runs) controls. **c)** Linear association between the areas deactivated during dorsal raphe photoactivation and structural connectivity maps for the default-mode, salience, and lateral cortical networks established relative to the ACA, aAI and MOp seeds, respectively. A strong association is observed between the brain-wide optogenetic response and the salience network (Tracer_aAI_ ~ CBV response : Coefficient = 0.20 [0.09, 0.30]), along with a weaker association to the default-mode network (Tracer_ACA_ ~ CBV response : Coefficient = 0.14 [0.04, 0.23]). Partial overlap with the default-mode network is potentially due to the lack of selectivity for the dorsal raphe, and the inclusion of other raphe nuclei during the photostimulation, such as the central raphe, structurally associated with the default-mode network. For details about the dataset and corresponding histology, see [[4]](https://paperpile.com/c/qBPnxE/RAR8). **d)** Photoactivation of CaMKII-positive neurons in the anterior insular area leads to a strong association with the structural connectivity of the salience network (Tracer_aAI_ ~ BOLD response: Coefficient = 0.24 [0.14, 0.35]).


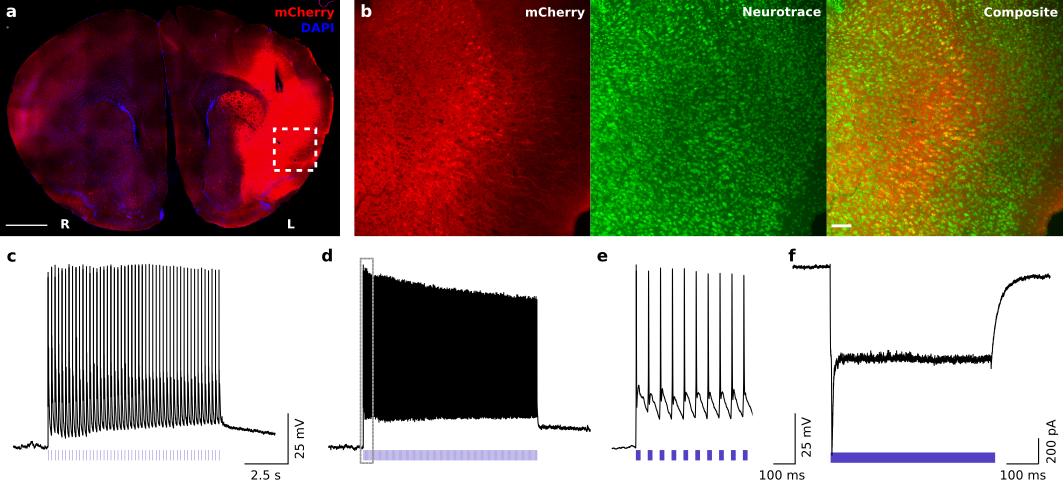


**Figure S8. a)** Histological slice showing the area of virus injection, expressing ChR2-mCherry fusion protein (red), and fiber implant site. Blue denotes cell nuclei (DAPI). The scale bar indicates 1 mm. **b)** Close-up on the insular area with Neurotrace as a marker for neurons (green), and composite image. The scale bar indicates 100 µm. Current-clamp electrophysiological recordings on an *ex vivo* brain slice in an mCherry-expressing neuron, with photostimulation at 5 Hz **(c)** and 20 Hz **(d, e)**. **f)** Voltage-clamp during prolonged (500 ms) photostimulation. Blue lines indicate photostimulation pulses.

1. [Gutierrez-Barragan D, Basson MA, Panzeri S, Gozzi A. Infraslow State Fluctuations Govern Spontaneous fMRI Network Dynamics. Curr Biol. 2019;29:2295–2306.e5.](http://paperpile.com/b/qBPnxE/ZjRd)

2. [Menon V. Large-scale brain networks and psychopathology: a unifying triple network model. Trends Cogn Sci. 2011;15:483–506.](http://paperpile.com/b/qBPnxE/JCPP)

3. [Grandjean J, Zerbi V, Balsters JH, Wenderoth N, Rudin M. Structural Basis of Large-Scale Functional Connectivity in the Mouse. J Neurosci. 2017;37:8092–8101.](http://paperpile.com/b/qBPnxE/6DnJ)

4. [Grandjean J, Corcoba A, Kahn MC, Upton AL, Deneris ES, Seifritz E, et al. A brain-wide functional map of the serotonergic responses to acute stress and fluoxetine. Nat Commun. 2019;10:350.](http://paperpile.com/b/qBPnxE/RAR8)
